# Toll-like receptor 4 in pancreatic damage and immune infiltration in acute pancreatitis

**DOI:** 10.3389/fimmu.2024.1362727

**Published:** 2024-03-22

**Authors:** Jordan Mattke, Carly M. Darden, Michael C. Lawrence, Jayachandra Kuncha, Yumna Ali Shah, Robert R. Kane, Bashoo Naziruddin

**Affiliations:** ^1^ Baylor University, Institute of Biomedical Studies, Waco, TX, United States; ^2^ Baylor University Medical Center, Annette C. and Harold C. Simmons Transplant Institute, Dallas, TX, United States; ^3^ Islet Cell Laboratory, Baylor Scott and White Research Institute, Dallas, TX, United States

**Keywords:** TLR4, pancreatitis, inflammation, macrophage, neutrophil, NET

## Abstract

Acute pancreatitis is a complex inflammatory disease resulting in extreme pain and can result in significant morbidity and mortality. It can be caused by several factors ranging from genetics, alcohol use, gall stones, and ductal obstruction caused by calcification or neutrophil extracellular traps. Acute pancreatitis is also characterized by immune cell infiltration of neutrophils and M1 macrophages. Toll-like receptor 4 (TLR4) is a pattern recognition receptor that has been noted to respond to endogenous ligands such as high mobility group box 1 (HMGB1) protein and or exogenous ligands such as lipopolysaccharide both of which can be present during the progression of acute pancreatitis. This receptor can be found on a variety of cell types from endothelial cells to resident and infiltrating immune cells leading to production of pro-inflammatory cytokines as well as immune cell activation and maturation resulting in the furthering of pancreatic damage during acute pancreatitis. In this review we will address the various mechanisms mediated by TLR4 in the advancement of acute pancreatitis and how targeting this receptor could lead to improved outcomes for patients suffering from this condition.

## 1 Introduction

The pancreas is an organ comprised of exocrine acinar tissue and endocrine islet tissue. Inflammation targeting the endocrine islets of the pancreas results in type 1 diabetes mellitus, which can be managed through the use of exogenous insulin to replace insulin production lost following beta cell destruction. Acute pancreatitis is an inflammatory condition within the pancreas characterized by damage to the organ in the form of exocrine acinar cell death and local and systemic inflammation (1). Acute pancreatitis may present in mild, moderate, severe forms. Mild pancreatitis presents with no local or systemic complications and no organ failure while moderate and severe pancreatitis both present with local and systemic complications and organ failure. Patients with acute pancreatitis present with epigastric pain radiating to the back, elevated serum amylase and lipase activity, and characteristics of pancreatitis based on ultrasonography, computed tomography, or magnetic resonance imaging (2, 3). The most common causes of acute pancreatitis are gallstones and alcohol, but other causes include hypertriglyceridemia, medication toxicity, trauma, hypercalcemia, various infections, autoimmune, ischemia, and hereditary causes (2). One of the other hallmarks of acute pancreatitis is the infiltration of immune cells such as neutrophils and macrophages following initial insult of pancreatitis, which generates further pancreatic damage (4, 5).

Although mortality due to pancreatitis has decreased over time, hospitalizations and cost of care have significantly increased for patients suffering from acute pancreatitis (6). It is also estimated that ~18% of patients presenting with acute pancreatitis will have recurrence or develop chronic pancreatitis (1). The current therapeutic strategies for managing acute pancreatitis include intravenous fluid resuscitation, nutritional support, and administration of analgesics (1). However, there is currently no targeted therapy for the treatment of acute pancreatitis.

Many cell types are implicated in the progression of acute pancreatitis. It is believed that acute pancreatitis is initiated by one of many factors that results in dysregulation of acinar cells resulting in the production of proinflammatory cytokines and chemokines (7). Following the induction of pancreatitis, neutrophils have been found to infiltrate the pancreas as soon as 1 hour after induction of pancreatitis (4). Proinflammatory M1 macrophages can be found infiltrating the pancreas during the early inflammatory stages of pancreatitis while M2 macrophages have been noted to be more prevalent during the resolution of phases of pancreatitis several days after pancreatitis induction (8). Infiltration of CD4+ T cells can be noted as soon as 6 hours following cerulein challenge in mice further contributing to pancreatic damage (9).

Toll-like receptors (TLRs) are the best characterized class of pattern recognition receptor. There are currently 11 recognized TLRs in humans while there are 13 TLRs in mice. These receptors can be found on the cell surface (TLR1, TLR2, TLR4, TLR5, TLR6, and TLR10) as well as intracellularly localized to endosomes (TLR3, TLR7, TLR8, TLR9, TLR11, TLR12, and TLR13) (10).

TLR1 associates with TLR2 to recognize mycobacterial lipoprotein as well as triacylated lipopeptides (11). TLR6 also forms a heterodimer with TLR2 recognizing macrophage-activating lipopeptide-2 and other diacylated lipopeptides (12). Although TLR1, TLR2, and TLR6 have been shown to be expressed on the surface, Motoi et al. have demonstrated that signaling of TLR1/TLR2 and TLR2/TLR6 actually happens in the endolysosomes (13). TLR5 is known to recognize bacterial flagellin and is also able to form a physical complex with TLR4 (14, 15). TLR10 is a unique toll-like receptor for several reasons. First, the ligands for TLR10 are currently unknown as it is a disrupted pseudogene in mice. TLR10 has been shown to be most homologous to TLR1 and TLR6 and is able to bind to the TLR1/2 ligand, PAM_3_CSK_4_ (16). TLR10 is also the only toll-like receptor to demonstrate anti-inflammatory properties (17). TLR4 is one of the most extensively studied TLRs, which responds to a variety of ligands and will be covered in more detail in the next section.

While the previously discussed toll-like receptors have been shown to the be localized to the plasma membrane of cells, several toll-like receptors have been shown to be localized to the endosomal compartments of cells. TLR3 recognizes and binds double-stranded RNA, which is usually the byproduct of viral infection (18). TLR7 and TLR8 recognize guanosine- and uridine-rich single-stranded RNA as well as imidazoquinoline-like molecules (19, 20). Imidazoquinoline is a tricyclic organic molecule known for enhancing immune responses to viruses and cancers (21, 22) TLR9 recognizes and is activated in response to microbial DNA sequences containing unmethylated CpG dinucleotides (23). Mouse TLR11 has been noted to recognize unknown components of uropathogenic bacteria and a profilin-like molecule of a protozoan parasite (10). TLR12 also plays a major role in the recognition of profilin of *Toxoplasma gondii* (24). TLR13 has been shown to be a key receptor for detecting bacterial RNA (25).

Bacterial translocation is a phenomenon which live bacteria that colonize the intestines or their products are able to cross the intestinal barrier into neighboring organs or the circulatory system resulting in remote organ inflammation and complications. This has been noted in both animal models and human trials during the progression of severe acute pancreatitis (26). Pancreatitis can also occur following viral infection with viral hepatitis, coxsackie and echoviruses, hemorrhagic fever viruses, CMV, and VZV being the most common (27). Because TLRs play a major role in bacterial and viral recognition, the activation of these receptors could play a protective role in the progression of pancreatitis following bacterial and viral infection.

Up to this point all of the discussed ligands for toll-like receptors are related to bacterial and viral infection and serve a role in inflammation in order to protect the body from infection. However, toll-like receptors are also able to respond to markers of sterile inflammation and cell apoptosis. For example, it has been demonstrated that mRNA released from necrotic cells stimulates the activation of TLR3 (28). High mobility group box 1 (HMGB1) released by innate immune cells in response to TNF or IL-1β is able to activate both TLR2 and TLR4 (29).

Heat shock proteins (HSP) are a class of protein originally found to be produced in response to a sudden increase in temperature. However, over time many different types of stresses have been associated with the upregulation of these proteins. These proteins fall into 3 main categories based on their size. HSP60 and HSP70 are both implicated in protein folding and unfolding as well as protein assembly while HSP90 has been shown to prevent steroid binding to DNA (30). Ethridge et al. demonstrated both HSP70 and HSP27 are significantly upregulated during the induction of pancreatitis in mice (31). Cao et al. also showed that HSP60 and HSP70 were significantly upregulated following the induction of pancreatitis in mice and the inhibition of p38 caused a decrease in expression of these heat shock proteins (32). In humans HSP60 serves to activate both TLR2 and TLR4 to promote proliferation of venous smooth muscle cells (33). HSP27 released after global ischemia is able to stimulate both TLR2 and TLR4 resulting in increased NF-ΚB signaling, which upregulates monocyte chemoattractant protein (MCP)-1 and intercellular adhesion molecule (ICAM)-1 as well as cytokine IL-6 (34).

## 2 Toll-like receptor 4 signaling and pathological relevance in acute pancreatitis

**Figure 1:**
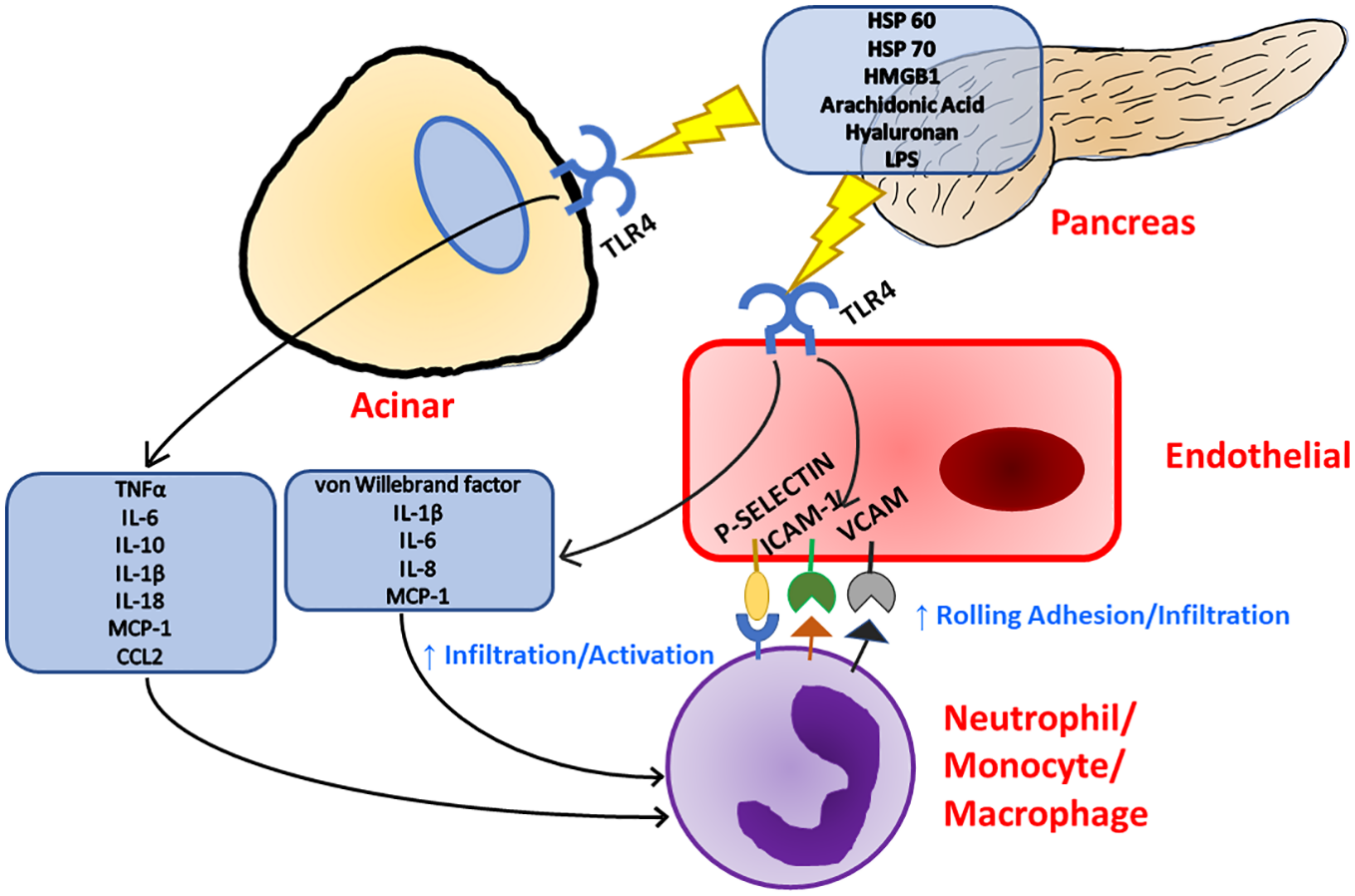
Inflammation in the pancreas can be initiated with TLR4 signaling as TLR4 has been known to respond to a multitude of ligands. Upon TLR4 stimulation, both acinar and endothelial cells release pro-inflammatory cytokines and chemokines which leads to immune cell recruitment and activation. Immune cell recruitment is further supported by TLR4 stimulation as endothelial cells upregulate the expression of surface adhesion molecules.

Toll-like receptor 4 (TLR4) is a receptor expressed on a multitude of cell types recognizing bacterial lipopolysaccharides (LPS), viral RNA, saturated fatty acids, and damage associated molecular patterns (DAMPs) such as high mobility group box 1 (HMGB1) protein and heat shock proteins 60 and 70 (**Figure 1**) (33, 35–39). HMGB1 has been found to be a key marker of many inflammatory conditions such as Alzheimer’s disease, Parkinson’s disease, and multiple sclerosis in the nervous system (40). HMGB1 is also implicated in inflammatory heart disease as well as well as several vascular inflammatory diseases (41, 42). HMGB1 has even been implicated in musculoskeletal diseases such as osteoarthritis (43). The production of HMGB1 and its signaling through TLR4 has been shown to be especially important in the progression of severe acute pancreatitis as administration of HMGB1 to mice resulted in increased pancreatic injury and NF-ΚB signaling that could be attenuated in TLR4-deficient mice (44). Pancreatitis patients with higher HMGB1 levels have also corresponded to increased disease severity (45). TLR4 can also be activated by pancreatic elastase, which has been shown to be elevated during acute pancreatitis (46). A comprehensive list of TLR4 ligands and their effects on different cell types within the pancreas is listed in **Table 1**.

**Table 1:** TLR4 responses to various ligands within pancreatic tissue.

Ligand	Cell Type	TLR4 Response	References
LPS	Acinar	Increased ROS, apoptosis, TNFα, IL-6, IL-10, IL-1β, IL-18, MCP-1	(50–53)
	Endothelial	Increased P-Selectin, VCAM1, IL-1β, IL-6, IL-8	(54, 55)
	Macrophages	M1 Polarization	(56)
	Neutrophil	Increased Survival	(57)
Arachidonic Acid	Acinar	Increased CCL2 and P-Selectin	(58)
HMGB1	Macrophages	Increased IL-8, TNF	(29)
	Neutrophil	Increased NADPH, ROS	(59, 60)
Hyaluronan	Endothelial	Increased IL-8	(61)
Heat Shock Protein 60	Vascular Smooth Muscle Endothelial Cells	Increased Migration, IL-8	(62)
	Macrophages	Increased TNFα, NO	(38)
Heat Shock Protein 70	Macrophages	Increased TNFα	(63)
Heat Shock Protein 27	Endothelial	Increased MCP-1, ICAM-1	(34)
Fatty Acid	Endothelial	Increased IL-6, IL-8, CCL5, CXCL10	(64)

**Figure 2:**
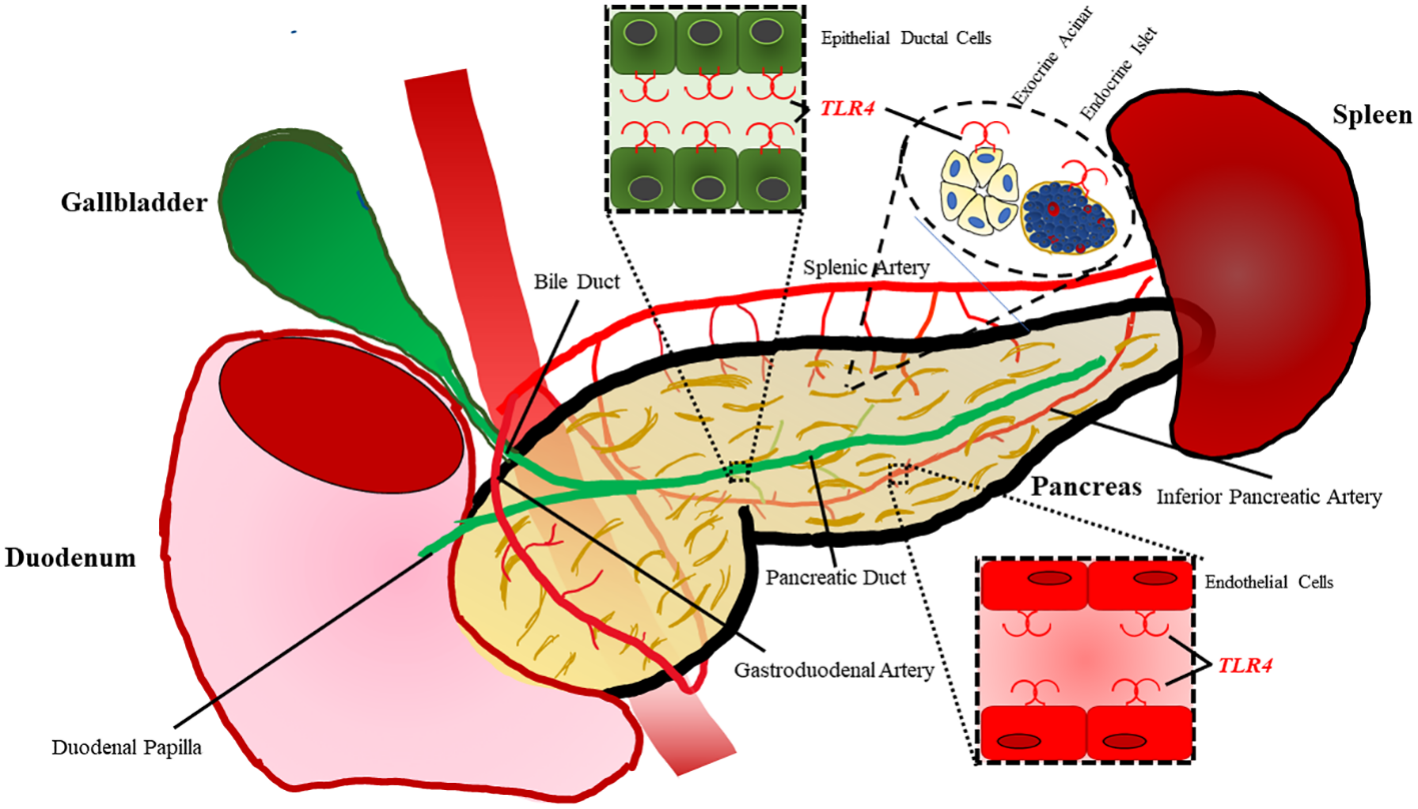
TLR4 is expressed on a variety of cell types within the pancreas. Epithelial cells lining the ductal tissue of the bile and main pancreatic duct are known to express TLR4. TLR4 is also expressed by exocrine acinar tissue as well as endocrine islets. The pancreas also has vasculature composed of endothelial cells with also have been shown to express TLR4.

Within the pancreas Li et al. demonstrated TLR4 is localized to pancreatic ductal epithelium, vascular endothelium, and islets while being absent in exocrine acinar cells of rats (**Figure 2**) (47). However, TLR4 signaling has proven to play a significant role in acinar cell inflammation during acute pancreatitis, which will be discussed in a later section. TLR4 expression is increased on monocytes 24 hours after the onset of acute pancreatitis and reduces to normal levels after 7 days (48). During severe acute pancreatitis, TLR4 is found to be elevated in liver, kidney, and intestinal tissue following induction of pancreatitis (49).

The process of TLR4 signaling by LPS begins with LPS binding protein (LBP) binding and escorting LPS to CD14. CD14 then transfers LPS to the TLR4-MD2 complex (65). Concentrations of LBP have been found to be significantly elevated in patients suffering from severe acute pancreatitis indicating the process of TLR4 signaling may have a role in systemic complications associated with severe acute pancreatitis (66, 67). Upon stimulation with LPS, TLR4-MD2 oligomerizes and is able to signal through the MyD88 dependent pathway to activate NF-κB and produce proinflammatory cytokines such as TNFα, IL-1β, IL-6, and IL12 (35). As markers such as IL-6 has proven to be significantly altered when looking at the severity of pancreatitis, TLR4 signaling could have a role in the progression of this disease (**Table 2**) (79). TLR4 is also able to signal through the MyD88 independent pathway through TRIF activating NF-κB and IRF3 to produce type I interferons (35). Inhibition of NF-κB signaling using Withaferin A has led to more beneficial outcomes in cerulein induced pancreatitis as leukocyte infiltration was decreased as well as fibrosis (80). Therefore, indirectly decreasing NF-κB signaling through inhibition of TLR4 could result in beneficial outcomes in the study of acute pancreatitis. An important downstream target of TLR4 signaling in pancreatitis is TRAF6, which is involved in both the TLR4 dependent and TLR4 independent pathways. This plays a significant role in the progression of pancreatitis as TLR4 deficient mice demonstrated much slower progression of pancreatic inflammation when compared to WT mice. TRAF6 was significantly higher in TLR4 deficient mice when compared to WT and TRAF6 was localized to pancreatic acinar cells (81). TRAF6 has also demonstrated a protective role in the progression of acute pancreatitis in acinar cells as stimulation of TLR4 lead to increased SOCS1 and SOCS3 expression, which are responsible for the degradation of TRAF6 through polyubiquitination. Knockout of TLR4 prevented the progression of acute edematous pancreatitis to acute necrotizing pancreatitis with the administration of cerulein and LPS (82).

**Table 2:** TLR4 related molecules used as diagnostic biomarkers.

Marker	Source	Findings	References
**IL-1β**	Mouse Pancreas	Neutrophils and macrophages are major producers of IL-1β	(68)
	Mouse Pancreas	IL-1β correlates with the severity of pancreatitis	(69)
	Human Serum	Associated with severity of disease	(70)
**IL-18**	Human Serum	Elevated with increased severity of pancreatitis	(71)
	Human Serum	Elevated with renal and respiratory failure during acute pancreatitis	(72)
	Human Serum	Significantly elevated in patients with complicated by pancreatic necrosis and remote organ failure	(73)
**IL-6**	Human Serum	Elevated in mild and severe acute pancreatitis	(74)
	Human Serum	Associated with severe disease	(70)
	Human Serum	Increased in severe acute pancreatitis starting 5 hours after onset	(75)
	Human Serum	Elevated in patients with acute pancreatitis complications	(76)
	Human Serum	Elevated in patients that did not survive	(45)
**IL-8**	Human Serum	Elevated in severe acute pancreatitis	(74)
	Human Serum	Elevated in patients that did not survive	(45)
**TNF**	Human Serum	Elevated in severe acute pancreatitis	(74)
**HMGB1**	Human Serum	Elevated in patients with severe acute pancreatitis	(77)
	Rat Pancreas and Serum	Elevated in pancreatic tissue 12 hours after induction of acute necrotizing pancreatitis. Significantly elevated in serum 12 hours after pancreatitis induction	(78)
	Human Serum	Elevated in patients that did not survive	(45)

**Figure 3:**
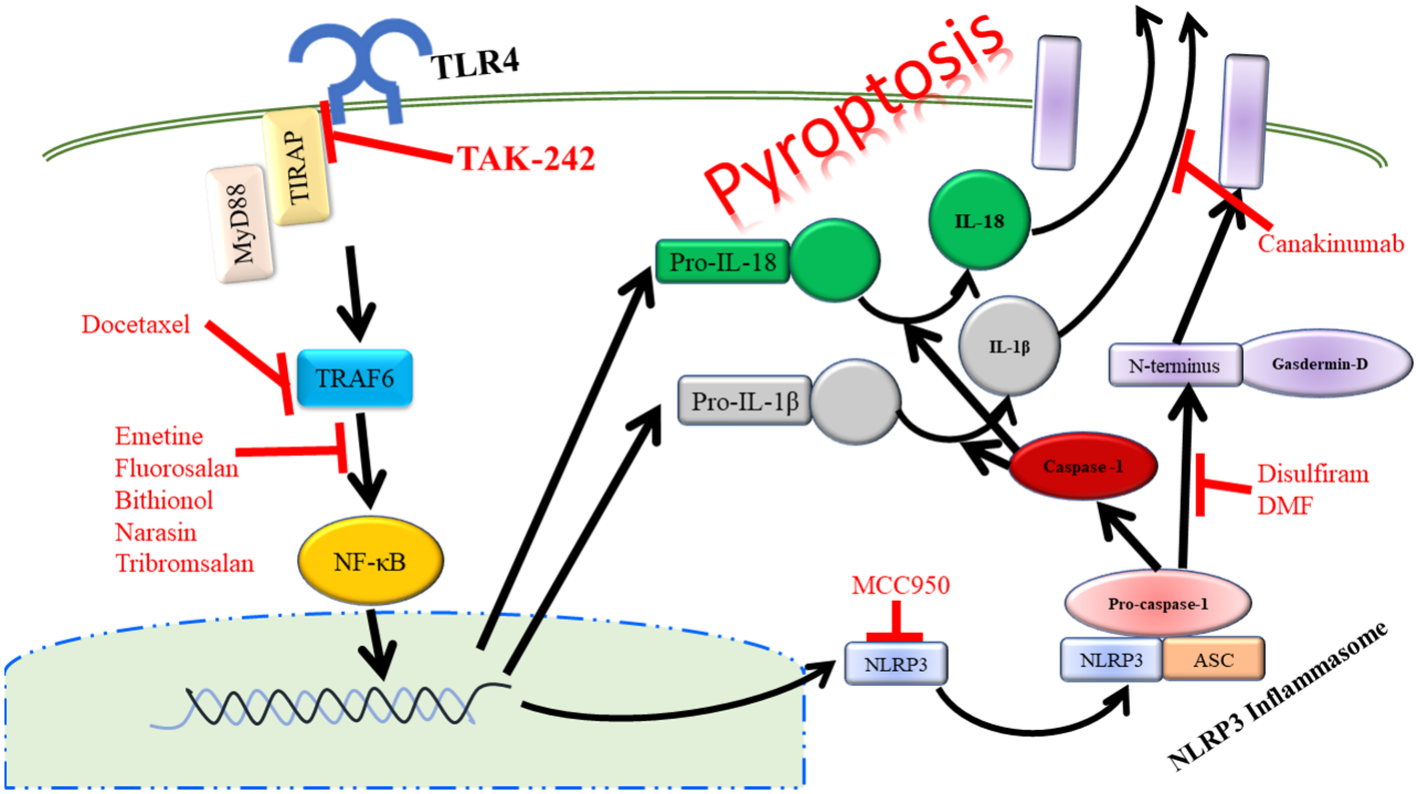
Activation of TLR4 can lead to the inflammatory process of pyropotosis. Many therapeutic drugs have been developed to target the different components of pyroptosis pathway.

Another key outcome of TLR4 signaling via NF-κB is the activation of the NOD-leucine rich repeat family pyrin domain containing protein 3 (NLRP3) inflammasome. Upon activation, NLRP3 forms a complex with ASC and pro-caspase-1. This complex formation and activation results in the conversion of pro-caspase-1 to active caspase-1, which will then cleave pro-IL-1β and IL-18 to active IL-1β and IL-18 that will be released from the cell promoting further inflammation. This process also results in the cleavage of gasdermin D. The N-terminal fragment of this cleavage will then form a pore in the plasma membrane allowing for further release of IL-1β and IL-18. This inflammatory process mediated by the NLRP3 inflammasome has been termed pyroptosis (**Figure 3**) (83). NLRP3, ASC, and caspase-1 are involved in inflammation associated with acute pancreatitis as loss of any of these signaling components has been shown to reduce the severity of acute pancreatitis (84). In a study focusing on patients with acute pancreatitis, it was demonstrated that higher serum IL-18 levels corresponded to pancreatitis complicated by pancreatic necrosis and remote organ failure showing that pyroptosis was increased in the organs of these patients (73). Targeting of the TLR4/NLRP3 axis with emodin, rhein, baicalin, and chrysin significantly reduced acinar cell necrosis and decreased nitric oxide production in macrophages demonstrating more beneficial outcomes in mice by targeting this pathway (85). NLRP3 is also a promising target in severe acute pancreatitis in that Sendler et al. propose that activation of this pathway in macrophages initiates innate inflammatory responses such as neutrophil recruitment and maturation as well as acts as a Th2-cell mediator for the adaptive immune system (86). It has also been shown that inhibition of caspase-1 leads to reduced acinar cell death by necrosis in severe acute pancreatitis (87). Therefore, targeting of TLR4 upstream of caspase-1 could at least partially inhibit cleaving and activation of caspase-1, which would result in more beneficial outcomes to those suffering from pancreatitis. Taken further, TLR4 on platelets can also signal through the NLRP3-ASC inflammasome leading to activation of caspase-1 promoting increased neutrophil extracellular trap (NET) formation leading to increased production of TLR4 agonist, S100A8/A9, which creates a positive feedback loop of inflammation during pyroptosis (88).

Another outcome of TLR4 signaling is the production of macrophage migration inhibitory factor (MIF), which has been shown to be a significant indicator of the severity of pancreatitis in both humans and mice (89–91). Inhibition of MIF has led to alleviated damage in pancreatic and renal tissues in a severe acute pancreatitis model through the attenuation of the NLRP3 pathway (92). MIF plays a key role in the NLRP3 inflammasome assembly leading the production and release of IL-1β in macrophages (93).

Activation of TLR4 can also lead to the activation of RIP3 and the induction of necroptosis (94). While apoptosis is usually associated with an orderly process of cell disassembly with little or no DAMP release, necroptosis is associated with massive release of DAMP molecules resulting in enhanced inflammation as a result of cell death (95). Inhibition of TLR4 signaling using small molecule inhibitor of, TAK-242, has led to attenuated necroptosis in an acute pancreatitis model as evidenced by a reduced expression of RIP3 within the pancreas of treated mice (96).

## 3 TLR4 in acinar cells

TLR4 plays a significant role in inflammation within acinar cells of the pancreas. Treatment with LPS has been shown to significantly increase the amount of apoptotic acinar cells in a cerulein induced pancreatitis (50). Upon stimulation with LPS, primary pancreatic acinar cells were found to have a significant increase in intracellular reactive oxygen species (ROS) as viability was decreased (51). LPS also induces apoptosis and expression of TNF-α, IL-1β, and IL-18 mRNA in AR4-2J cells (derived from azaserine-induced malignant nodules in rat pancreas) (52). Another study found that TLR4-positive acinar cells respond to LPS by activating the inflammasome and producing TNF-α, IL-6, IL-10, IL-1β, IL-18, and MCP-1 during acute pancreatitis, and these effects could be exacerbated by alcohol (**Figure 1**) (53). Vona-Davis et al. investigated the effects of LPS and TNFα treatment on AR4-2J cells and noted that there was an activation of both STAT3 and SOCS3 in response to this treatment with significant increases in SOCS3 expression with treatment of LPS and IL-6 while TNFα enhanced expression of STAT3, which also stimulated SOCS3 expression (97). This highlights the importance of SOCS3 in the resolution of inflammation and a possible role of TLR4 in not only the initiation of inflammation, but the resolution of inflammation within acinar cells.

Isolated pancreatic acini are able to respond to arachidonic acid via TLR4 resulting in the upregulation of monocyte chemoattractant protein 1 (CCL2) and P-selectin, which would cause increased immune recruitment and adhesion in the pancreas (58). In a study carried out by Sztefko and Panek it was noted that elevated levels of arachidonic acid could be involved in the development of complications in acute pancreatitis (98). *In vivo* TLR4^-/-^ and CD14^-/-^ mice showed reduced acinar atrophy in a severe acute pancreatitis model (99). Small molecule inhibitor of TLR4, TAK-242, increased the viability of pancreatic acinar cells, decreased lactate dehydrogenase, and reduced apoptotic cell death following exposure to taurocholate. This was accompanied by decreased release of cytochrome c into the cytoplasm, reduced mitochondrial swelling, and decreased mitochondrial Ca^2+^ buffering capacity following exposure to taurocholate (100). Administration of natural product, biochanin A, resulted in reduced pancreas damage through decreased TLR4 and NLRP3 signaling (101). This is further evidenced in a study that showed knockout of NLRP3 or gasdermin D in acinar tissue of cerulein treated mice resulted in reduced pyroptosis (102). In TLR4 deficient mice there is significantly less acinar cell necrosis (103).

## 4 TLR4 in endothelial cells

TLR4 plays a key role in the inflammation of endothelial cells, which could contribute to the infiltration of immune cells during the progression of acute pancreatitis. First, stimulation of TLR4 in endothelial cells leads to significant increases in surface adhesion proteins as well as increases in P-selectin and von Willebrand factor expression (**Figure 1**) (104). P-selectin plays a significant role in the adhesion and infiltration of neutrophils as inhibition of P-selectin has resulted in reduced damage and neutrophil infiltration in experimental pancreatitis (105, 106). Elevated von Willebrand factor has also proven to be a useful biomarker for severe acute necrotizing pancreatitis (107). TLR4 signaling in endothelial cells can lead to Weibel-Palade body degranulation, NF-κB activation, and vaso-occlusion (108). It has been further demonstrated that TLR4 participates in HLA class I signaling to upregulate P-selectin and von Willebrand factor expression leading to increased rolling adhesion and infiltration by monocytes (109). Vein endothelial cells have been shown to respond to LPS via TLR4 signaling to produce cytokines such as IL-1β, IL-6, and IL-8 further contributing to immune infiltration and activation (54). Increased IL-8 concentrations in serum are correlated to more complicated pancreatitis as well as neutrophil elastase (110). These results implicate serum IL-8 as a biomarker for neutrophil activation leading to pancreatitis complications. This also shows that reduction in production of IL-8 through the inhibition of TLR4 signaling could result in improved pancreatitis outcomes.

Within the pancreas, intraperitoneal administration of LPS lead to significant increases in P-selectin and vascular cell adhesion molecule-1 (VCAM-1) expression within the pancreas (55). As VCAM-1 and P-selectin are both associated with increased immune cell adhesion and infiltration, a reduction of these markers through inhibition of TLR4 signaling could serve as a therapeutic strategy to attenuate damage due to immune cell infiltration and activation. TLR4 of endothelial cells is also able to sense components of the extracellular matrix such as hyaluronan to trigger inflammation and the initial stages of would defense and repair (61). As hyaluronan has been shown to be accumulated in the edematous interstium during acute pancreatitis, this could lead to the activation of TLR4 in endothelial cells further contributing to inflammation and immune cell recruitment to the pancreas (111).

## 5 TLR4 in macrophages

**Figure 4:**
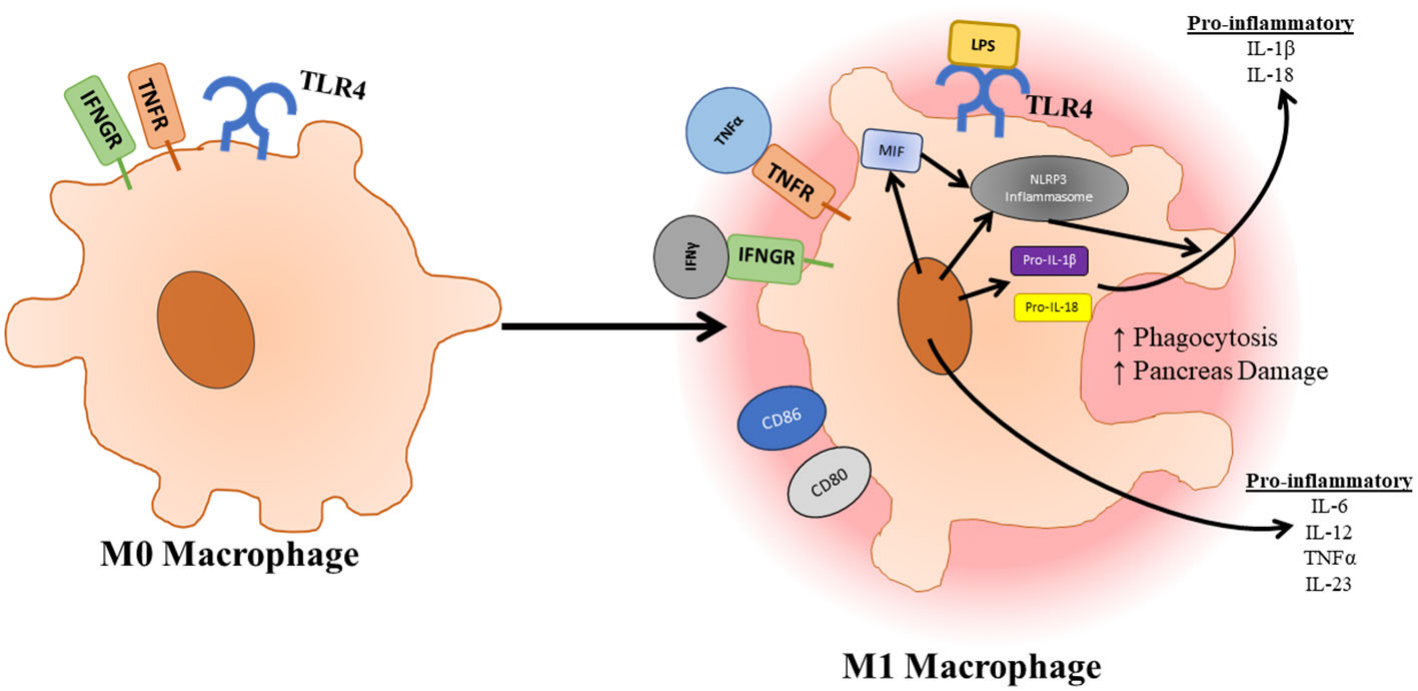
Once macrophages have infiltrated the pancreas, they can be activated and polarized to an M1 state through TLR4 signaling. Activation of macrophages is further supported by factors released by pancreatic tissue. Upon activation, polarized macrophages increase their phagocytic capacity as well as release pro-inflammatory cytokines which further immune infiltration and destruction.

TLR4 and the associated pathways are essential to the maturation and polarization of macrophages (**Figure 4**). Macrophages can be divided into different categories based on stimulus. M0 macrophages are non-activated macrophages, which can differentiate into proinflammatory M1 macrophages or anti-inflammatory M2 macrophages. M1 macrophages develop following exposure to inflammatory signals such as IFNγ, LPS, or TNFα, which activates STAT1, NF-κB, p65/p50, and IRF5 resulting in the production of IL-6, TNFα, IL-23, and iNOS. M2 macrophages can be further subdivided into M2a, M2b, M2c, and M2d subtypes all of which play a role in resolution of inflammation and wound healing and form in response to different stimuli (56). As mentioned previously, the activation of TLR4 not only signals through TRAF6 to promote inflammation and survival signals, but it also signals through SOCS1 and SOCS3 to provide negative feedback for this process. SOCS1 has proven to be especially important in dampening immune infiltration and inflammation responses as mice deficient in SOCS1 showed increases in inducible nitric oxide synthase expression in the pancreases of these mice, which appeared to preferentially damage exocrine over endocrine tissue (112). Qin et al. also show that deficiency of SOCS3 in macrophages resulted in higher levels of M1 macrophage genes (113). However, this contrasts to a study carried out by Gordon et al. who found that silencing of SOCS3 promoted a shift of activated M1 macrophage markers to increased expression of M2 macrophage markers. This same study also noted that silencing of SOCS3 resulted in increased phagocytic capacity of these macrophages (114). In a study conducted by Arnold et al. focusing on renal and peritoneal inflammation, SOCS3 expression correlated strongly with disease severity and renal injury. SOCS3 was also shown to colocalize with markers of M1 macrophage polarization (115). The role of SOCS3 in the activation of macrophages in pancreatitis is further supported by a study showing that macrophage-specific deletion of SOCS3 developed less severe pancreatitis and produced less TNFα in response to cerulein injections. This same study also highlighted the importance of CCL2 in the migration of macrophages from the bone marrow to the pancreas during the induction of pancreatitis (116). This further emphasizes the importance of TLR4 response in acinar cells as CCL2 has been found to be upregulated by TLR4 in response to arachidonic acid. NLRP3 signaling also plays a large role in the activation of macrophages and associated pro-inflammatory as well as the compensatory anti-inflammatory responses. In a study where bone marrow-derived macrophages were incubated with pancreatic acini, macrophages were found to secrete increased IL-1β and IL-18 showing an upregulation of the NLRP3 pathway. In a study conducted by Sendler et al. NLRP3 activation was shown to have a significant role in both the hyperinflammation responses as macrophages are activated promoting enhanced recruitment and activation of other immune cells. Inhibition of the NLRP3 inflammasome using MCC950 resulted in significantly reduced neutrophil infiltration, T cell activation, and disease severity in mice. They also showed that NLRP3 signaling has a role in the anti-inflammatory response syndrome in which Th2 and Tregs are activated by IL-18 in the absence of IL-12, which results in enhanced pancreatic fibrosis and permits bacterial translocation into pancreatic necrosis or severe sepsis (86). Since TLR4 is known to activate the NLRP3 inflammasome, inhibition of TLR4 could also lead to some of these beneficial effects.

Macrophages play an important role in the progression of pancreatitis as a delicate balance between M1 and M2 macrophages is needed in order for pathogen clearing and wound healing to occur. During acute pancreatitis, infiltrating macrophages are mainly activated and differentiated into an M1 phenotype (5). This is further confirmed by Sendler et al. who showed M2 macrophages were found in non-necrotic areas of pancreatic tissue while M1 macrophages were found in necrotic fields during induction of pancreatitis. Co-culture of bone marrow derived macrophages with acinar cells showed significant increases in IL-6 and TNFα as well as anti-inflammatory IL-10 (86). TLR4 signaling plays a large role in the differentiation of M1 macrophages. In macrophages advanced glycation end products have been found to stimulate TLR4 signaling resulting in an M1 phenotype, which could be inhibited by treatment with TAK-242 (117). The fungal protein paracoccin has also been found to stimulate TLR4 resulting in a pro-inflammatory M1 phenotype (118). Treatment of macrophages with berberine results in a reduction in the production of inflammatory factors and reduced polarization of macrophages to an M1 phenotype by inhibiting the binding of MyD88 to TLR4 (119). Blocking of TLR4 and TNFR1 promotes of a shift of macrophages to an M2 phenotype (120). Therefore, inhibition of TLR4 could serve as a therapeutic strategy to dampen inflammation induced by macrophages by promoting a shift from inflammatory to anti-inflammatory macrophages.

Administration of pancreatic elastase results in increased inflammatory responses in THP-1 cells mediated by TLR4 (46, 121). High fat diet in acute pancreatitis rats aggravated infiltration of activated, inflammatory macrophages by signaling of TLR4 which could be inhibited by the administration of TAK-242 (96). Carbon monoxide releasing molecule has been found to inhibit TLR4 signaling in macrophages leading to reduced production of TNFα and proinflammatory cytokines in cerulein-induced pancreatitis (122).

## 6 TLR4 in neutrophils

TLR4 signaling plays a significant role in the activation and lifespan of neutrophils. Stimulation of TLR4 using purified LPS almost completely prevents neutrophil apoptosis at early timepoints by signaling through NF-κB (57). TLR4 also signals through MEK kinase leading to the inhibition of G-protein-coupled receptor kinases 2 and 5, which are responsible for the internalization or surface chemokine receptor CXCR2, resulting in increased neutrophil activation and migration (123). TLR4 signaling is also unique in neutrophils in that neutrophils only utilize the MyD88-dependent signaling pathway when stimulated with ligands such as LPS (124). HMGB1 during hemorrhagic shock/resuscitation results in increased TLR4 signaling resulting in increased NADPH oxidase activity in neutrophils leading to an increased production of reactive oxygen species (59). The activity of NADPH oxidase and production of reactive oxygen species by neutrophils results in increased TLR2 and ICAM-1 expression on endothelial cells, which creates a positive feedback loop of inflammation and cellular adhesion (125). High fat diet in acute pancreatitis induced rats results in increased activation and infiltration of neutrophils into the pancreas by signaling through TLR4 (96). TLR4^-/-^ mice have demonstrated significantly less neutrophil infiltration in an acute pancreatitis model (103, 126).

One unique feature of neutrophils is their ability to produce web-like structures composed of decondensed chromatin fragments wrapped in histones, proteases, granules, and cytoplasmic proteins referred to as neutrophil extracellular traps (NETs) (**Figure 4**) (127). NETs have been found to play a significant role in the progression of a taurocholate induced acute pancreatitis model as neutrophil depletion and administration of DNase I lead to attenuated pancreas damage (128). In patients with acute pancreatitis increases in platelet microparticles were observed. When these platelet microparticles were mixed with healthy neutrophils, there was an increase in myeloperoxidase, neutrophil elastase, and histone H3 production as well as the release of NETs. This indicates that the production of NETs also plays a role in the progression of pancreatitis in humans (129). TLR4 on platelets induces platelet binding to neutrophils resulting in neutrophil activation and NET formation (130). The presence of free radicals has been shown to play a role in the progression of pancreatitis and the production of NETs (131). These free radicals such as superoxide can lead to an increased production of NETs at the site of sterile inflammation through signaling of TLR4 (132). Treatment of neutrophils with HMGB1 (elevated during severe acute pancreatitis) results in increased production of NETs through increased signaling of TLR4 (60). The production of NETs is also supported by the activation of the NLRP3 inflammasome under sterile conditions as NLRP3 was found to support both nuclear envelope and plasma membrane rupture during the release of NETs (133). Not only are NETs produced directly through TLR4 signaling, but NET release is also supported by cytokines produced in other tissues during the progression of acute pancreatitis. IL-8 (elevated following TLR4 signaling in endothelial cells) promotes the production of NETs through its interaction with CXCR2, which is upregulated on neutrophils in response to TLR4 signaling (134). IL-1β (upregulated in response to TLR4 signaling in acinar and endothelial cells in response to TLR4 signaling)has also shown the ability to stimulate the production of NETs in neutrophils as well (135).

## 7 TLR4 in remote organ complications during severe acute pancreatitis

TLR4 has a demonstrated role in pancreatic inflammation and in the progression of acute pancreatitis. In addition, previous studies have also demonstrated a role for TLR4 in remote organ complications as well. It has long been recognized that severe acute pancreatitis can lead to lung damage, which in turn leads to increased mortality of patients with pancreatitis (136). During the induction of acute pancreatitis mice utilizing LPS, an upregulation of both TLR4 and MIF were observed in the lungs of treated mice. The inhibition or loss of MIF resulted in lower TLR4 expression in the lungs of mice following the induction of pancreatitis as well as increased survival outcomes further implicating TLR4 in respiratory distress following the onset of pancreatitis (137). TLR4^-/-^ mice also showed significantly less lung myeloperoxidase activity following the cerulein induction of pancreatitis (99). Induction of pancreatitis in rats using L-arginine also resulted in increased serum creatinine and BUN indicating increased renal injury during this induction of pancreatitis. This same study also showed increased liver biomarkers aspartate transaminase (AST) and alanine transaminase (ALT) during the induction of pancreatitis. Down regulation of HMGB1/TLR4/NF-ΚB signaling using protocatechuic acid resulted in significant reduction in levels of pancreatic amylase and lipase as well as reduced AST, ALT, creatinine, and BUN (138). The trends of reduced renal and hepatic damage were also noted in TLR4 deficient mice as well as significantly lower serum levels of interleukin-1 and tumor necrosis factor (49). Taken together, not only could inhibition of TLR4 affect immune infiltration and damage within the pancreas during the onset of acute pancreatitis, but inhibition of TLR4 could also alleviate remote organ complications during severe acute pancreatitis such as adult respiratory distress syndrome.

## 8 Conclusions and future directions

**Figure 5:**
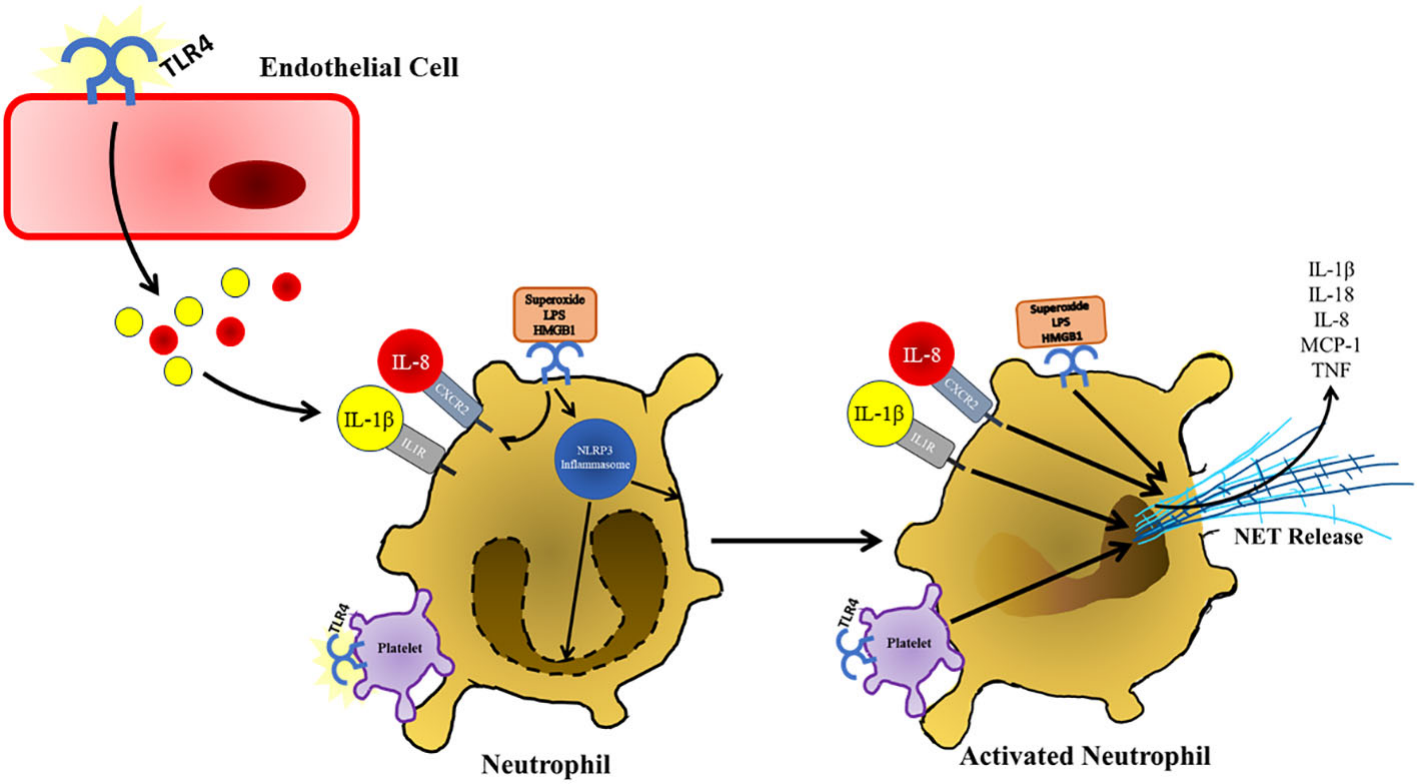
Following TLR4 stimulation and other damage associated signals present in the pancreas, neutrophils respond by secreting NETs, which further contribute to pancreatic damage and immune infiltration during the progression of acute pancreatitis. NET formation is also supported by TLR4 activation and binding of platelets. TLR4 stimulation also supports the production of NETs by upregulating of CXCL2, which is stimulated by IL-8 that presents at increased concentrations during the progression of acute pancreatitis. NETs are also released in response to increased IL-1β, which is also increased in response to TLR4 stimulation in various cell types in response to TLR4 signaling.

TLR4 is a pattern recognition receptor expressed on a diverse population of cells and plays a multitude of roles in the progression in acute pancreatitis. The initial insult of pancreatitis results in the production of key ligands for TLR4 such as HMGB1 and heat shock proteins associated with sterile inflammation. These ligands can then stimulate localized inflammation in acinar and endothelial tissue through TLR4 to promote the production of many inflammatory factors which increase infiltration and activation of innate immune cells (**Figure 1**). Once these innate immune cells enter the pancreas, TLR4 signaling stimulates the polarization of macrophages to an inflammatory M1 phenotype, which will lead to further destruction of exocrine tissue as well as continued infiltration of immune cells (**Figure 4**). However, further study is warranted in the area of TLR4 signaling and the polarization of macrophages as overproduction of M2 macrophages can result in chronic pancreatitis (139). Clarification of the role of SOCS3 and TLR4 in macrophage polarization is also of interest in the progression of pancreatitis as deletion of SOCS3 resulted in more beneficial outcomes following induction of pancreatitis. SOCS3 also colocalized with M1 macrophage markers. However, conflicting information states that SOCS3 deficiency results in increased M1 gene expression. TLR4 also has a significant role in the infiltration and activation of neutrophils as TLR4 deficient mice show reduced neutrophil infiltration and pancreas damage. TLR4 has a role in the production of NETs, which are shown to be increased in the progression of pancreatitis and play a role in destruction of exocrine tissue (**Figure 5**). We would also like to acknowledge that very little research has been carried out investigated TLR4 signaling in ductal epithelial cells. TLR4 has been identified in cells of the ductal epithelium of the pancreas (47). As TLR4 has been shown to be upregulated in epithelial cells of the bowel during inflammatory conditions such as Crohn’s disease and ulcerative colitis, it would also be worth investigating TLR4 expression and signaling of the pancreatic ductal epithelial cells during the progression of acute pancreatitis as this could also contribute to immune cell infiltration and activation (140). Investigation of TLR4 inhibition has been shown to be clinically feasible as small molecule inhibitor of TLR4, TAK-242, has been used in a clinical trial for use in the treatment of sepsis and has proven to be safe for use in humans (141). Because of the diversity of roles TLR4 plays in the progression of pancreatitis and pancreas damage, the inhibition of TLR4 could be worth investigating for the treatment of acute pancreatitis in humans.

## Author contributions

JM: Conceptualization, Investigation, Writing – original draft, Writing – review & editing. CD: Writing – review & editing. ML: Writing – review & editing. JK: Writing – review & editing. YA: Writing – review & editing. BK: Writing – review & editing. BN: Writing – review & editing, Conceptualization.
